# Antidepressant Effects of *Bifidobacterium animalis* CP-9 in LPS-Induced Depressive Mice

**DOI:** 10.3390/foods14244289

**Published:** 2025-12-12

**Authors:** Shenglan Su, Ziyi Jiang, Fang He, Cailing Chen, Yuping Yang, Liang Dong, Youhua Ren, Ke Li, Zongjun Li, Yuanliang Wang

**Affiliations:** 1 College of Food Science and Technology, Hunan Agricultural University, Changsha 410128, China; sushenglan@stu.hunau.edu.cn (S.S.); 15674979678@163.com (Z.J.); fanghe_0615@163.com (F.H.);; 2 Ausnutria Dairy (China) Company Ltd., Changsha 410200, China; 3 Inner Mongolia Kangxin Food Co., Ltd., Hohhot 010000, China; 4 Shenzhen Siyomicro Bio-tech Co., Ltd., Shenzhen 518116, China; 5 Hunan Province Key Laboratory of Food Science and Biotechnology, Changsha 410128, China

**Keywords:** *Bifidobacterium animalis* CP-9, depression-like behavior, gut–brain axis, inflammatory response, gut microbiota

## Abstract

Depression is a common mood disorder, and growing evidence has revealed the critical role of gut microbiota in its onset and progression. The gut–brain axis, which connects the central nervous system and intestinal microecology, offers new strategies for depression intervention. In this study, an acute depression model was established in mice using lipopolysaccharide (LPS), and the potential antidepressant effects and mechanisms of *Bifidobacterium animalis CP-9* were investigated. The results indicated that CP-9 may exert antidepressant effects through multiple pathways, including modulation of peripheral and central inflammatory responses, restoration of gut microbiota balance, enhancement of short-chain fatty acid (SCFA) production, and regulation of neurotransmitter metabolism such as γ-aminobutyric acid (GABA) and 5-hydroxytryptamine (5-HT). Notably, intervention with CP-9 at a dose of 10^8^ CFU/mL significantly alleviated depressive-like behaviors in mice, suggesting its promising potential in the prevention and treatment of mood disorders.

## 1. Introduction

Depression is one of the most prevalent psychiatric disorders worldwide, characterized by persistent low mood, maladaptive responses to environmental stimuli, and often accompanied by cognitive impairment and social dysfunction [1]. Chronic stress is a major pathogenic factor, capable of inducing neurodegenerative changes in key brain regions such as the hippocampus and prefrontal cortex. These neuroplasticity impairments are considered fundamental biological mechanisms underlying the disease [2,3]. Current evidence suggests that depression results from the interaction between genetic susceptibility and environmental stress, mediated by neurotransmitter imbalance and other complex pathological processes [3,4].

The global health burden of depression has shown a marked upward trend. The Global Burden of Disease Study (2020) reported that depression accounts for nearly 10% of disability-adjusted life years (DALYs), ranking as the second largest health threat after cardiovascular diseases [5]. More concerning is the sharp rise in incidence among adolescents: recent epidemiological data indicate a nearly 40% increase in the prevalence of depression among individuals aged 12–18 years compared to 2010. Contributing factors may include academic stress, excessive screen exposure, and circadian rhythm disruption [6].

Although selective serotonin reuptake inhibitors (SSRIs) remain the primary clinical treatment for depression and are effective in improving core symptoms, their side effects (such as gastrointestinal discomfort, sleep disturbances, and sexual dysfunction) often reduce patient compliance [7,8]. Moreover, conventional treatments mainly provide symptomatic relief without fundamentally reversing neuroplastic damage or stress-induced pathology, and therapeutic responses vary greatly among individuals. Some patients remain unresponsive to existing therapies, underscoring the urgent need for novel interventions based on alternative mechanisms.

Probiotics, defined as live microorganisms with specific metabolic and regulatory functions, have emerged as promising candidates due to their ability to restore gut microbial balance via competitive colonization, secretion of bioactive metabolites, and immune regulation. Recent studies have shown that certain strains, such as *Lactobacillus plantarum* PS128 and *Bifidobacterium longum* NCC3001, exert significant effects through the gut–brain axis. Mechanisms include the enhancement of short-chain fatty acid (SCFA) synthesis (e.g., butyrate upregulating hippocampal BDNF expression to promote neurogenesis) [9], modulation of tryptophan metabolism with a 17–23% increase in serotonin precursor conversion [10], and elevated synthesis of neurotransmitters such as GABA and norepinephrine [11].

Among the various probiotic strains with psychobiotic potential, *Bifidobacterium animalis* CP-9, the focus of this study, presents distinctive metabolic and anti-inflammatory properties based on preliminary evidence. Unlike some commonly studied strains, CP-9 has been shown in vitro and in prior animal models to more effectively promote the production of beneficial metabolites like butyrate and to exhibit superior systemic and central anti-inflammatory capacity, particularly in downregulating key pro-inflammatory cytokines such as IL-6 and TNF-α. However, a systematic evaluation of its efficacy in depression models, its behavioral impacts, and its hippocampal neuroprotective effects remains insufficient, and its unique mechanisms of action are yet to be fully elucidated. Building on this evidence, the present study employed an LPS-induced depressive mouse model to investigate the potential of *Bifidobacterium animalis* CP-9 as an antidepressant intervention. Behavioral tests (sucrose preference, forced swim), inflammatory cytokine assays (IL-6, TNF-α), and hippocampal histopathology were systematically evaluated to elucidate the mechanisms of CP-9 action. The findings are expected to provide experimental evidence for the translational development of CP-9 as a psychobiotic candidate for mood disorder prevention and treatment.

## 2. Materials and Methods

### 2.1. Materials

*Bifidobacterium animalis CP-9* was preserved in the Microbiology Laboratory of Hunan Agricultural University. Lipopolysaccharide (LPS, from *Proteus mirabilis*, ≥99%, Cat. No. L8880) and fluoxetine (≥98%, CAS: 54910-89-3) were purchased from Beijing Solarbio Science & Technology Co., Ltd., Beijing, China.

### 2.2. Animals

Seventy-two SPF-grade male BALB/cA mice (6–8 weeks, 18–22 g) were obtained from Hunan Slack Jingda Laboratory Animal Co., Ltd. (Changsha, Hunan Province, China License No. SCXK [Xiang] 2021-0002). The animals were housed at 22 ± 2 °C under a 12 h light/dark cycle with free access to food and water. All experimental procedures complied with the international guidelines for the care and use of laboratory animals and were approved under relevant pharmacology and toxicology regulations.

### 2.3. Preparation of LPS Solution

The LPS stock solution was prepared by dissolving 10.0 mg of LPS in sterile saline in a 100 mL volumetric flask, followed by vortex mixing until fully suspended. Sterile saline was added to the calibration line to yield a final concentration of 100 μg/mL. A total of 10.0 mL of this stock solution was diluted to 500 mL to prepare the working solution, yielding an application concentration of 2 mg/kg. Parallel dilution ensured consistency across preparations [12].

### 2.4. Establishment of the Depression Model

After a 21-day acclimatization period, the mice were using simple randomization divided into six groups (n = 6 per group): a blank control group, an LPS model group, a positive control group (fluoxetine), and CP-9 low-dose (10^7^ CFU/mL), medium-dose (10^8^ CFU/mL), and high-dose (10^9^ CFU/mL) groups. Mice in the treatment groups received a daily oral gavage of either CP-9 suspension or fluoxetine (0.1 mL/g), while the control groups were administered an equal volume of sterile saline. The intervention lasted for five weeks. Upon completion of the intervention period, mice were intraperitoneally injected with 0.2 mL of LPS (2 mg/kg) or sterile saline 60 ± 5 min after the final gavage [13].

### 2.5. Behavioral Tests

Sucrose Preference Test: Prior to the test, mice were acclimated to the sugar water for 1 h. Two 500 mL water bottles were placed in each cage, one containing 2% sucrose solution and the other containing plain water. To prevent position preference, the bottle positions were swapped every 12 h. The consumption of both sucrose solution and plain water was recorded. Sucrose preference (%) was calculated as: [Sucrose consumption/(Sucrose consumption + Water consumption)] × 100% [14].

Forced Swim Test: Mice were individually placed in a transparent cylindrical container (14 cm in diameter, 20 cm in height) filled with distilled water to a depth of 10 cm (water temperature maintained at 23 ± 1 °C). A 6-min timer was started immediately upon the mouse entering the water. The first 2 min served as an acclimatization period. The behavior during the final 4 min was video-recorded. The duration of immobility was quantified, defined as the passive floating state where the animal ceased struggling and made only minimal movements necessary to keep its head above water [15].

### 2.6. Confirmation of Modeling Success

Four hours after LPS injection, mice were subjected to behavioral testing. The sucrose preference test, forced swim test, and tail suspension test were used to evaluate depressive-like behavior [16].

### 2.7. Histopathological Sections of Tissue

Upon completion of the behavioral tests, three mice were randomly selected from each group using a standard random number table method. The selected mice were anesthetized with an intraperitoneal injection of sodium pentobarbital (30 mg/kg). Following anesthesia, the animals were euthanized by cervical dislocation. The skull was carefully opened to expose the brain, from which the entire brain tissue was rapidly and completely excised. The harvested brains were immediately placed in pre-cooled physiological saline to remove the superficial vascular membranes. Subsequently, the brain tissues underwent a series of procedures including gradient dehydration, paraffin embedding, and sectioning. The morphological characteristics of the hippocampal tissues were finally examined under an optical microscope [17].

### 2.8. Pro-Inflammatory Cytokine Assays

After behavioral experiments, mice were anesthetized with 3% sodium pentobarbital (30 mg/kg, i.p.) and sacrificed by cervical dislocation. The hippocampus was rapidly isolated, snap-frozen in liquid nitrogen, and stored at −80 °C for long-term preservation. Samples were aliquoted into pre-chilled cryotubes labeled with unique identifiers. For analysis, hippocampal tissues were weighed, and 90 μL of pre-cooled PBS buffer was added per 10 mg of tissue. Homogenates were centrifuged at 2000 r/min for 20 min at 4 °C (radius 8 cm). Supernatants were collected and the concentrations of IL-1β, IL-6, and TNF-α in hippocampal tissues were determined strictly according to the instructions of the ELISA kits (purchased from Shanghai Enzyme-linked Biotechnology Co., Ltd., Shanghai, China) [18].

### 2.9. Gut Microbiota 16S rRNA Gene Sequencing

After dissection, intestinal contents were collected, frozen on dry ice, and subjected to 16S rRNA sequencing (Wuhan Servicebio Technology Co., Ltd., Wuhan, China). Total DNA was extracted and amplified using primers targeting conserved regions, with sequencing adapters ligated to primer ends. PCR products were purified, quantified, and normalized to construct sequencing libraries. After quality control, libraries were sequenced using the Illumina NovaSeq 6000 platform (Illumina, San Diego, CA, USA). Raw image data were processed through base calling to generate sequenced reads, which were subsequently subjected to bioinformatics analyses [19].

### 2.10. Non-Targeted Metabolomics of CP-9

Non-targeted metabolomics of *Bifidobacterium animalis* CP-9 was performed using LC–MS (Panomix Biomedical Technology Co., Ltd., Suzhou, China). Raw mass spectrometry files were converted to mzXML format using the MSConvert tool in ProteoWizard (v3.0.8789). Data processing, including peak detection, filtering, and alignment, was conducted using the XCMS package in R with the following parameters: bw = 2, ppm = 15, peakwidth = c (5, 30), mzwid = 0.015, mzdiff = 0.01, method = “centWave”. Quantification was normalized to total peak area to correct for system bias. Metabolite identification was performed by matching parent ion (*m*/*z*) and fragment ion features to public databases (HMDB, MassBank, LipidMaps, mzCloud, KEGG) and an in-house library, with a mass error tolerance of ≤30 ppm [20].

### 2.11. Data Analysis

All statistical analyses were performed using SPSS 17.0 and GraphPad Prism (version 9.0; GraphPad Software, San Diego, CA, USA). Data are presented as mean ± standard deviation (SD).

Normality and homogeneity of variances were assessed before parametric testing. The Shapiro–Wilk test confirmed normality for all datasets (*p* > 0.05). Levene’s test indicated homogeneity of variances (*p* > 0.05), allowing the use of parametric tests.

For multiple-group comparisons (e.g., behavioral tests, cytokine levels, alpha diversity indices), one-way analysis of variance (ANOVA) was applied. When a significant main effect was found (*p* < 0.05), Tukey’s honestly significant difference (HSD) post-hoc test was used for pairwise comparisons. Exact *p*-values are reported throughout the results. Effect sizes for ANOVA are reported as partial eta squared (ηp^2^), interpreted as small (≥0.01), medium (≥0.06), and large (≥0.14).

For multivariate analyses of metabolomics and microbiota data, principal component analysis (PCA), partial least squares-discriminant analysis (PLS-DA), and orthogonal partial least squares-discriminant analysis (OPLS-DA) were performed using the ropls package (version 1.6.2) in R (version 4.3.0). Differential metabolites were identified based on a combination of variable importance in projection (VIP) > 1.0 from the OPLS-DA model and *p* < 0.05 from Student’s t-test (with false discovery rate (FDR) correction for multiple comparisons where applicable). KEGG pathway enrichment analysis was conducted via the MetaboAnalyst 5.0 platform. The significance of pathway enrichment was assessed using a hypergeometric test, and pathway impact was evaluated based on topological centrality.

## 3. Results

### 3.1. General Observations of Mice

After the acclimatization period, mice exhibited normal physiological states, characterized by glossy fur, high activity levels, good appetite, and normal excretion. Following acute depression induction with LPS, mice in the model group displayed dull and ruffled fur, localized piloerection, curled postures (limb retraction, arched back), reduced spontaneous activity, and diminished responsiveness to external stimuli. In contrast, fluoxetine-treated mice (positive control) and those in the CP-9 groups (C1, C2, C3) exhibited notable improvements compared with the model group. Their fur became smoother and shinier, grooming behaviors reappeared, postures were more relaxed, activity levels increased, resting time decreased, and responsiveness and exploratory behaviors were restored. These results indicate that *Bifidobacterium animalis* CP-9 effectively alleviated LPS-induced depressive-like behaviors and abnormal appearances in mice.

### 3.2. Behavioral Assessments

#### 3.2.1. Sucrose Preference Test

The sucrose preference index was calculated as:


$$\mathrm{Sucrose}\mathrm{Preference}\;(\%)=\frac{\mathrm{Sucrose}\mathrm{intake}}{\mathrm{Sucrose}\mathrm{intake}+\mathrm{Water}\mathrm{intake}}\times 100.$$

Data were normally distributed (Shapiro–Wilk *p* > 0.05) and variances were homogeneous (Levene’s *p* = 0.12). One-way ANOVA revealed a significant main effect of group (F (5,12) = 18.37, *p* < 0.001, ηp^2^ = 0.88).

As shown in Table 1, the model group showed a significantly lower sucrose preference index compared to the blank control group (*p* < 0.001). CP-9 intervention showed a dose-dependent effect. The medium-dose group (C2) showed the highest sucrose preference, significantly different from the model group (*p* = 0.007). The high-dose group (C3) did not differ significantly from the model group (*p* = 0.312) [21,22].

#### 3.2.2. Forced Swim Test

As shown in Table 2, the immobility ratio in the model group (41%) was significantly higher than that in the blank control group (28%) (*p* < 0.01), indicating successful induction of the despair phenotype characteristic of depressive-like behavior. CP-9 intervention exhibited a non-monotonic dose–response effect. In the low-dose group (C1), the immobility ratio was 0.37 ± 0.11, which was not significantly different from the model group (*p* > 0.05), suggesting that low-dose intervention did not surpass the threshold required for effective microbiota modulation. In the medium-dose group (C2), the immobility ratio decreased to 0.34 ± 0.03, showing significant improvement compared with the model group (*p* < 0.05), potentially associated with SCFA-mediated suppression of neuroinflammation. The high-dose group (C3) showed the most pronounced effect, with an immobility ratio of 0.27 ± 0.04, representing a 34.1% reduction compared with the model group (*p* < 0.01). This effect may be attributed to butyrate-driven enhancement of hippocampal BDNF signaling. Notably, the high dose (10^9^ CFU/mL) demonstrated the most potent effect in reducing despair-like behavior, which diverged from its subdued effect in the sucrose preference test. This discrepancy highlights the complexity of dose-dependent effects across different depressive-like behavioral domains [23].

### 3.3. Experimental Results of Inflammatory Factor Detection

The results of inflammatory factor detection are shown in Table 3. The model group exhibited significantly higher levels of IL-1β, IL-6, and TNF-α compared to the blank group (*p* < 0.01), indicating that the depression model successfully induced systemic inflammatory responses.

In the positive control group, IL-1β (168.15 vs. 376.54) was significantly lower than that in the model group (*p* < 0.01) but still significantly higher than that in the blank group (*p* < 0.01), suggesting that the drug partially suppressed inflammation. IL-6 levels returned to levels comparable to the blank group (*p* > 0.05), indicating that the drug had a specific inhibitory effect on the IL-6 pathway. TNF-α was significantly lower than that in the model group (*p* < 0.01) but higher than that in the blank group (*p* < 0.01), reflecting the limited regulatory effect of the drug on TNF-α.

Regarding the dose-dependent anti-inflammatory effects of Bifidobacterium, the C1 group (low dose) showed significantly lower levels of IL-1β, IL-6, and TNF-α compared to the model group (*p* < 0.01), with IL-6 even lower than that in the blank group (*p* < 0.05). The C2 group (medium dose) exhibited IL-1β and TNF-α levels close to those of the blank group, while IL-6 showed no significant difference from the blank group. The C3 group (high dose) demonstrated significantly lower IL-1β and TNF-α levels compared to the model group (*p* < 0.01), with IL-6 significantly lower than that in the blank group (*p* < 0.01). These results indicate that the low-dose CP-9 already had a significant anti-inflammatory effect but caused excessive downregulation of IL-6. The medium dose achieved a good anti-inflammatory effect without over-suppression, while the high dose exhibited a strong anti-inflammatory effect. Overall, the medium-dose group demonstrated the most balanced anti-inflammatory effect, particularly in normalizing IL-6 levels without over-suppression. The low and high doses, while effective in reducing overall inflammation, induced a significant under-shoot of IL-6, indicating a potential bimodal immunomodulatory effect that warrants further investigation.

**Figure 1:**
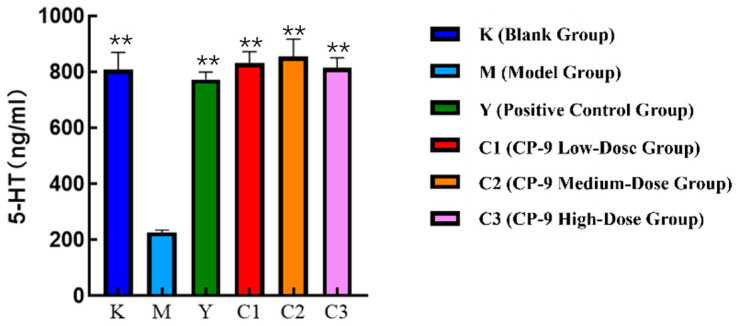
Experimental results of 5-HT detection for symptom factors. Note: ** *p* < 0.01 vs. model group.

The experimental results of the inflammatory factor 5-HT detection are shown in Figure 1. Compared with the blank group, the serum 5-HT level in the depression model group was significantly decreased (*p* < 0.01). Compared with the depression model group, the fluoxetine group (positive group) and all CP-9 dose groups showed increased serum 5-HT levels (*p* < 0.01). The 5-HT level was highest in the medium-dose CP-9 group, and the efficacy of all CP-9 dose groups was comparable to that of the fluoxetine group.

### 3.4. Hippocampal Histopathological Results

**Figure 2:**
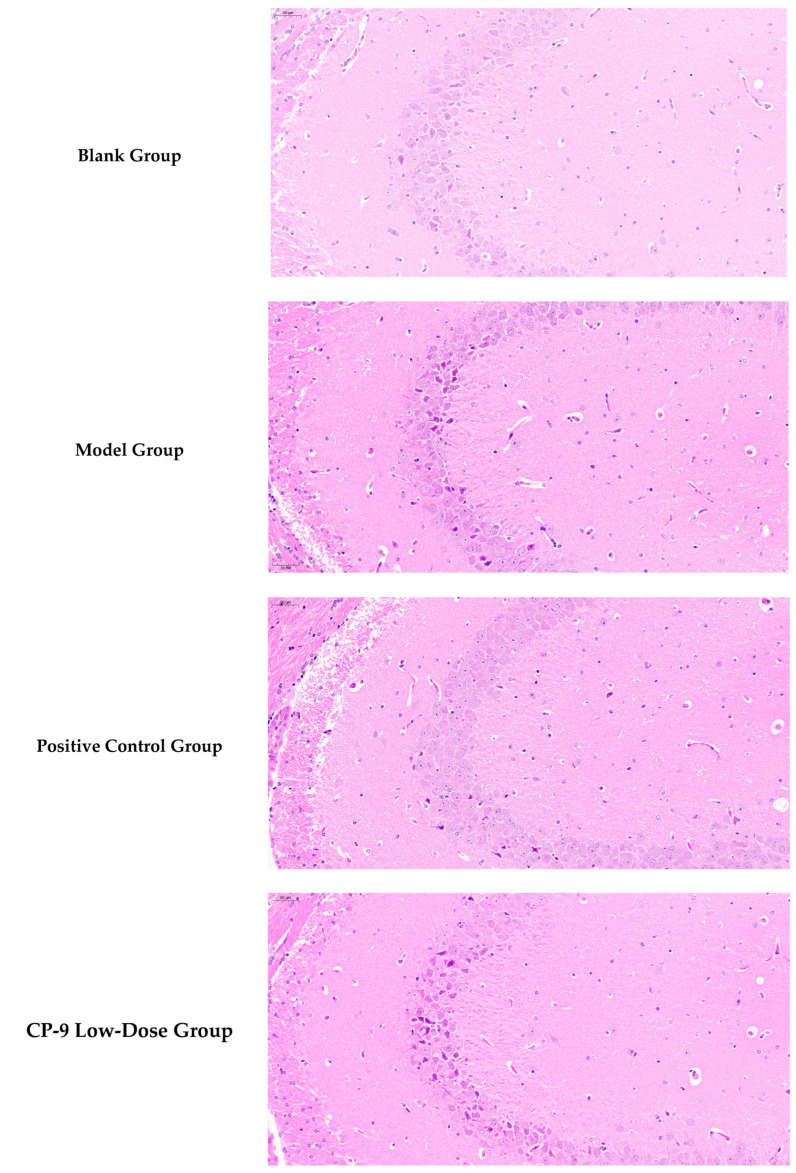

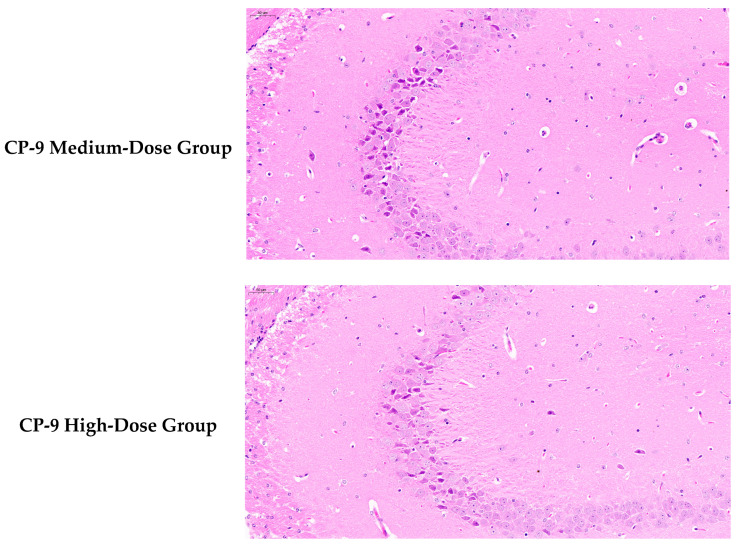
Mouse Hippocampal Tissue Slices.

As shown in Figure 2 based on blinded image analysis of six mouse hippocampal tissue sections, the experimental results are as follows: The blank group exhibited intact hippocampal cell structure with orderly arrangement. In contrast, the model group showed pyknotic nuclei, disordered neuronal arrangement, cell atrophy, reduced cell numbers, and significant glial cell hyperplasia, indicating neuroinflammation. Compared to the model group, the positive control group demonstrated a marked reduction in degenerated cells and significant restoration of neuronal structure.

The low-, medium-, and high-dose CP-9 groups showed significant improvements over the model group in multiple histological indicators, including neuronal density, glial cell count, degeneration index, and pathological score. These groups effectively reversed the model-induced neuronal loss and glial over-proliferation, and reduced tissue degeneration and pathological scores. In summary, CP-9, within the tested dose range, effectively suppressed neuropathological changes associated with the depression model, demonstrating potential therapeutic value.

### 3.5. Analysis of Gut Microbiota 16S rRNA Gene Amplicon Sequencing

The statistical results of Alpha diversity indices are shown in Table 4. The Chao1 index estimates the number of OTUs in a community, representing species richness, while the Shannon index reflects species diversity; a higher value indicates greater microbial diversity. As shown in Table 4, the Shannon and Chao1 indices in the depression model group (Group M) were significantly lower than those in the blank control group (Group K) (*p* < 0.05), indicating that depression induction led to a decrease in gut microbiota diversity and species richness.

**Figure 3:**
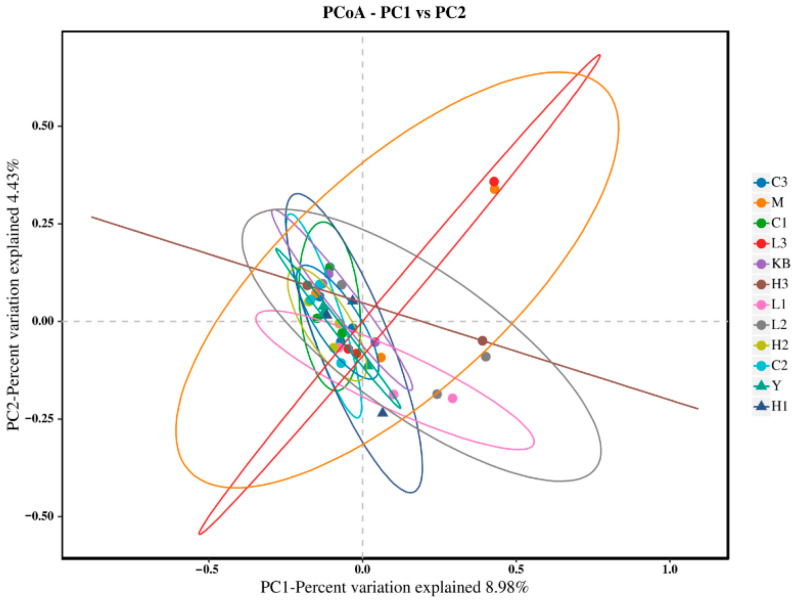
PCoA analysis plot.

**Figure 4:**
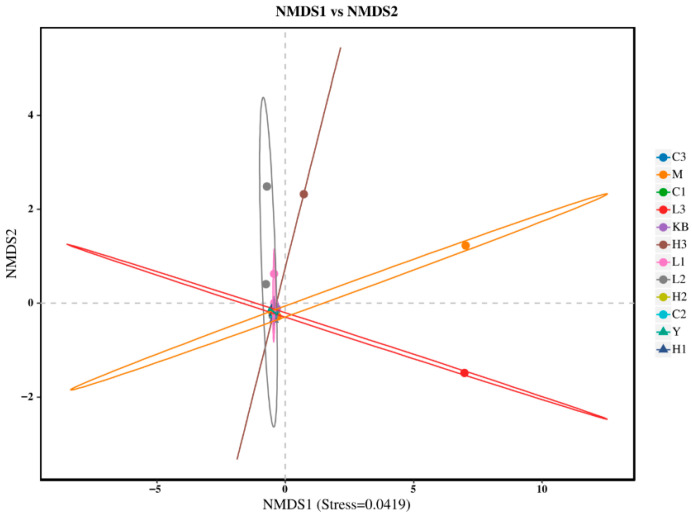
NMDS ordination plot.

The Shannon index in the high-dose group (Group C3) was close to that of Group K. In the PCoA analysis (Figure 3), Group C3 showed significant separation from Group M based on Bray-Curtis distance (*p* < 0.05) and clustered closer to Group K, suggesting that high-dose Bifidobacterium could partially reverse the microbial dysbiosis. Furthermore, NMDS analysis (Figure 4, Stress < 0.1) revealed that the samples from the CP-9 intervention groups (Group C) clustered progressively closer to Group K with increasing dosage, confirming a dose-dependent effect on the restoration of microbial community structure.

At the phylum level, the Firmicutes/Bacteroidetes (F/B) ratio, which is associated with depression, was significantly increased in the model group. In contrast, the F/B ratio gradually decreased in the intervention groups (Group C3: 1.3 vs. Group K: 1.0). The result from the positive control group (Group Y: 1.2) verified the effectiveness of the intervention.

### 3.6. Analysis of Gut Microbiota at the Genus Level in Mice

**Figure 5:**
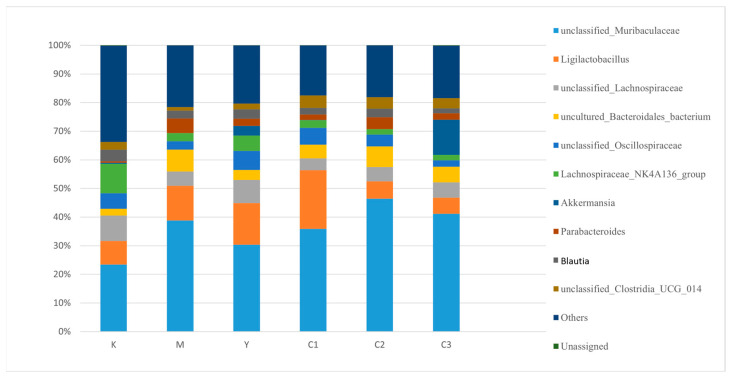
Genus-level species distribution bar chart.

Analysis of Key Taxa at the Genus Level is shown in Figure 5.

Significant microbial dysbiosis was observed in the Model group (Group M):

Compared to Group K, the abundance of unclassified_Muribaculaceae was significantly decreased in the feces of Group M mice, while the abundances of genera such as Ligilactobacillus, unclassified_Lachnospiraceae, and Parabacteroides were significantly increased. This indicates a structural imbalance of the gut microbiota under depressive conditions.

A notable restoration trend was observed in the gavage intervention groups:

With increasing doses of CP-9 (C1 → C2 → C3), the gut microbial structure in the intervention groups gradually shifted towards that of Group K. Group C3 (the high-dose group) exhibited the most significant restorative effect, characterized by a marked increase in the abundance of unclassified_Muribaculaceae, effective reductions in the abundances of Ligilactobacillus and unclassified_Lachnospiraceae, and improvements in both gut microbial diversity and homeostasis.

Regulatory effects on specific genera:

The abundance of Akkermansia decreased in Group M and was restored to a certain extent in the CP-9 intervention groups (C groups), suggesting that CP-9 may have a potential role in promoting gut barrier repair.

The abundance of *Parabacteroides*, which was elevated in the Model group, decreased progressively following CP-9 intervention.

In conclusion, gavage with *Bifidobacterium animalis* CP-9 effectively alleviated the gut microbiota dysbiosis in depressed mice, exhibiting a degree of dose-dependency, with the high-dose group showing the optimal effect. These findings provide potential microecological support for its antidepressant mechanisms.

### 3.7. Non-Targeted Metabolomics Data of CP-9

**Figure 6:**
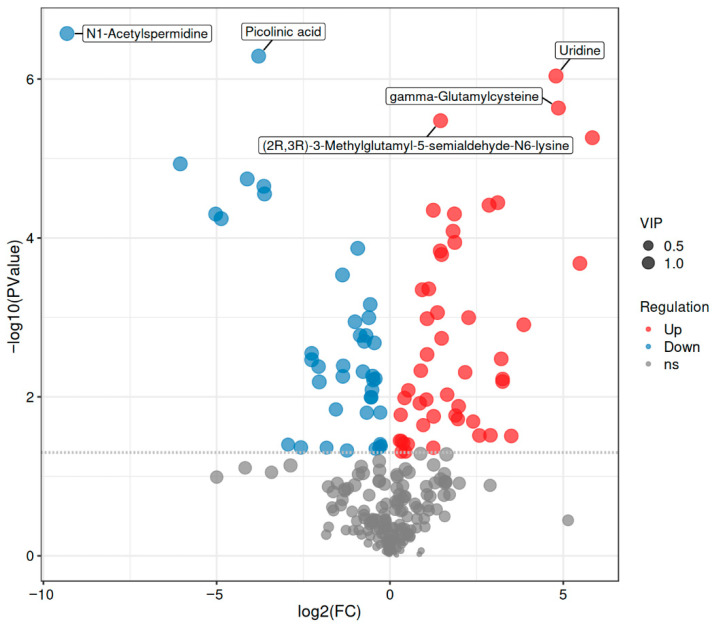
Volcano Plot of Differential Metabolites. Note: The size of the points represents the magnitude of the Variable Importance in Projection (VIP) score. Red points indicate differentially upregulated metabolites, blue points indicate differentially downregulated metabolites, and gray points represent metabolites that did not meet the criteria for differential significance. PCA and OPLS-DA models revealed clear separation between the CP-9 intervention and model groups, indicating distinct metabolic profiles. KEGG pathway analysis further demonstrated that differentially expressed metabolites were enriched in pathways such as phenylalanine metabolism, ABC transporters, and the TCA cycle, supporting the involvement of amino acid metabolism, energy homeostasis, and transmembrane transport in the antidepressant mechanisms of CP-9.

Based on non-targeted metabolomics analysis (Table 5 and Figure 6), a total of 89 statistically significant differential metabolites were identified in the CP-9 intervention group compared to the control group (48 upregulated and 41 downregulated). Further metabolic pathway analysis revealed that these differential metabolites were primarily involved in key biological processes such as amino acid metabolism (e.g., tryptophan/phenylalanine metabolism), neurotransmitter synthesis (e.g., GABAergic and monoaminergic pathways), energy metabolism, and inflammatory responses. These findings suggest that CP-9 may exert its antidepressant effects by synergistically regulating multiple metabolic networks, including neurotransmitter homeostasis, mitochondrial function, and neuroinflammatory responses.

Overall, the metabolomic analysis revealed significant alterations in several key metabolites associated with central nervous system (CNS) function. Notably, γ-aminobutyric acid (GABA), a major inhibitory neurotransmitter, was significantly upregulated in the CP-9 intervention group (log_2_FC = 0.5, *p* = 5.46 × 10^−3^), suggesting enhanced GABAergic signaling. Conversely, 2-phenylethanol was downregulated (log_2_FC = −0.48, *p* = 6.14 × 10^−3^), which may reflect shifts in aromatic amino acid metabolism. These changes align with the observed behavioral improvements and indicate that CP-9 modulates neurotransmitter homeostasis. Conversely, metabolites such as cytosine and L-2,4-diaminobutyric acid were significantly upregulated (log_2_FC = 1.87 and 2.17, *p* = 1.14 × 10^−4^ and 4.91 × 10^−3^), suggesting their potential involvement in regulating neuronal metabolic activities and amino acid synthesis processes, thereby contributing to the maintenance of nervous system homeostasis.

Volcano plot analysis (Figure 4) further validated the screening results of differential metabolites. As shown in Figure 4, the distribution pattern of metabolites based on log_2_(FC) and −log_10_(P) revealed distinct differential expression characteristics. Red markers (upregulated), blue markers (downregulated), and gray markers (non-significant) represent different expression trends, while the circle size is positively correlated with the variable importance in projection (VIP) value of the metabolites. Notably, metabolites such as N1-acetylspermidine, picolinic acid, uridine, and gamma-glutamylcysteine not only exhibited significant differential expression (*p* < 0.05, |log_2_FC| > 1) but also possessed high VIP values (VIP > 1), indicating their potential as key biomarkers involved in the pharmacological effects of CP-9.

In summary, CP-9 intervention induced systemic changes in multiple metabolites, particularly those involved in neuroregulatory pathways. Its antidepressant effects may be achieved through the coordinated regulation of neurotransmitter metabolism, redox balance, and inflammatory responses. This study provides critical metabolomic evidence for further investigation into functional mechanisms and clinical translation applications.

### 3.8. KEGG Metabolic Pathway Analysis of CP-9

**Figure 7:**
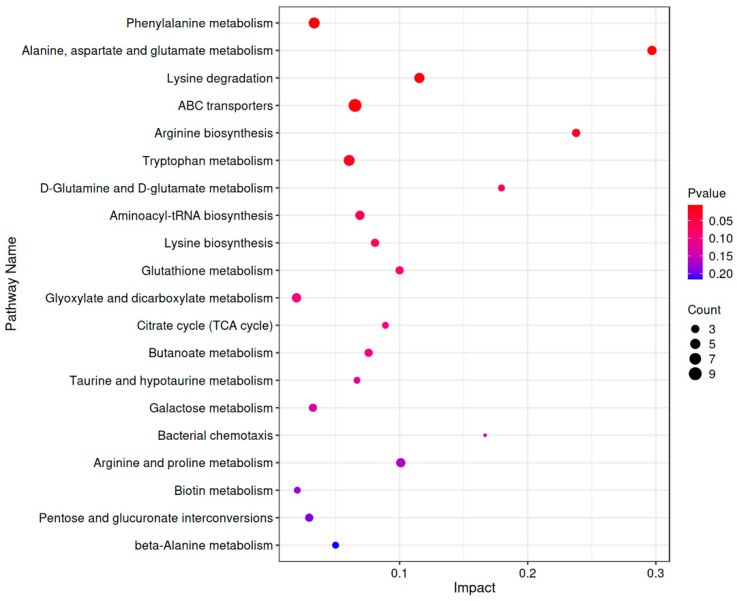
Bubble chart of metabolic pathway impact factors. Note: The horizontal axis represents the Impact value of metabolites enriched in different metabolic pathways, while the vertical axis lists the enriched pathways. The size of the points corresponds to the number of metabolites mapped to each pathway. The color of the points is related to the *p*-value, with redder colors indicating smaller *p*-values and bluer colors indicating larger *p*-values.

As shown in Figure 7, KEGG enrichment analysis revealed that the differential metabolites induced by CP-9 intervention were significantly enriched in multiple key metabolic pathways (*p* < 0.01), primarily including:

Phenylalanine metabolism; Alanine, aspartate, and glutamate metabolism; Lysine degradation; Arginine biosynthesis; ABC transporter pathways. Among these, the phenylalanine metabolism pathway contained 6 differential metabolites (*p* = 5.32 × 10^−3^, Impact = 0.0333), while the alanine, aspartate, and glutamate metabolism pathway included 4 differential metabolites (*p* = 6.41 × 10^−3^, Impact = 0.297). These findings suggest that aberrant amino acid metabolism may play a critical role in the antidepressant effects of CP-9.

Furthermore, the metabolomic analysis revealed significant alterations in the ABC transporter pathway (involving 9 differential metabolites, *p* = 1.18 × 10^−2^), which is closely associated with transmembrane transport and energy metabolism, potentially playing an important role in drug metabolism and cellular homeostasis regulation. The lysine degradation pathway (6 metabolites, *p* = 8.32 × 10^−3^, Impact = 0.210) and arginine biosynthesis pathway (6 metabolites, *p* = 1.01 × 10^−2^, Impact = 0.0705) also showed notable enrichment, further supporting the potential involvement of multiple amino acid metabolic pathways in regulating emotional behavior and neural function.

Notably, key pathways such as tryptophan metabolism (5 metabolites, *p* = 9.25 × 10^−3^), D-glutamate and D-aspartate metabolism, glutathione metabolism, and the tricarboxylic acid (TCA) cycle were also enriched to varying degrees. These pathways are closely related to oxidative stress, energy supply, and neurotransmitter synthesis, suggesting that CP-9 may exert its antidepressant effects through multi-dimensional metabolic regulatory mechanisms.

In summary, this study reveals the synergistic regulation of multiple metabolic pathways under CP-9 intervention, providing a theoretical foundation for further elucidating its mechanisms of action.

## 4. Discussion

Numerous previous studies have demonstrated that Bifidobacterium species exert antidepressant effects through multiple pathways. Wang et al. [24] reported that Bifidobacterium breve alleviated depressive behaviors in chronically stressed mice by promoting the synthesis of 5-hydroxytryptophan (5-HTP) in the gut, thereby increasing brain 5-HT concentrations. Luo et al. [25] found that, similar to fluoxetine, Bifidobacterium increased monoamine neurotransmitter levels (NE, 5-HT, DA) in brain regions such as the prefrontal cortex and hippocampus in rat models. Other studies have shown that Bifidobacterium can reverse chronic stress-induced hyperactivity of the hypothalamic-pituitary-adrenal (HPA) axis by suppressing corticotropin-releasing hormone (CRH) and serum corticosterone [26]. In terms of gut microbiota and barrier function restoration, Bifidobacterium intervention restored gut microbiota diversity in depressed mice, increased the abundance of beneficial bacteria (e.g., *Lactobacillus* and *Akkermansia*), reduced harmful bacteria (e.g., *Clostridium*), and improved intestinal physiological barrier damage by repairing small intestinal villi length and enhancing mucus layer integrity [24,26]. Additionally, regarding metabolite-mediated mechanisms, short-chain fatty acids (SCFAs) such as propionate and butyrate produced by Bifidobacterium metabolism activate G protein-coupled receptors (GPRs), regulating neuroendocrine and immune responses and suppressing inflammation [27,28]. Bifidobacterium strains with high GABA production alleviated corticosterone-induced neuronal damage and improved depression by enhancing inhibitory neurotransmission [29].

In this study, a lipopolysaccharide (LPS)-induced acute depression mouse model was used to systematically evaluate the antidepressant potential and mechanisms of *Bifidobacterium animalis* CP-9. The results demonstrated that CP-9 intervention significantly reversed LPS-induced depressive-like behaviors, as evidenced by increased sucrose preference index and reduced immobility time in the forced swim test. The medium-dose group (10^8^ CFU/mL) showed the most optimal effects in behavioral improvement and inflammatory factor regulation. Inflammatory factor detection results indicated that CP-9 effectively suppressed the abnormal elevation of pro-inflammatory cytokines (IL-1β, IL-6, TNF-α) in the hippocampus and serum, mitigating systemic inflammatory responses. Gut microecological analysis revealed that CP-9 intervention significantly restored gut microbiota diversity in mice, increased the abundance of beneficial bacteria such as Bifidobacterium and Lactobacillus, reduced the proportion of the pathogenic Escherichia-Shigella, and normalized the Firmicutes/Bacteroidetes ratio. Although the high-dose group (C3) exhibited more pronounced effects in anti-inflammatory and gut microecological restoration, its efficacy in some behavioral indicators was attenuated, suggesting that CP-9 may exhibit dose-dependent and pathway-specific differences.

Based on non-targeted metabolomics data and a comprehensive analysis of behavioral, inflammatory, and gut microbiota changes, it is hypothesized that *Bifidobacterium animalis* CP-9 alleviates depressive-like behaviors and neuroinflammation through the following synergistic mechanisms:

Anti-inflammatory Effects Mediated by Short-Chain Fatty Acids (SCFAs). Compared to the depression model group, the CP-9 intervention group showed significant enrichment of metabolites in short-chain fatty acid (SCFA) metabolic pathways, such as propionate and butyrate. As critical metabolic signaling molecules, SCFAs can activate G protein-coupled receptors (GPR41, GPR43) in the gut and central nervous system, effectively inhibiting the release of pro-inflammatory cytokines and suppressing NF-κB signaling pathway activation, thereby alleviating neuroinflammatory responses and protecting neuronal integrity [30,31].

Regulation of γ-Aminobutyric Acid (GABA) Levels and Remodeling of Neurotransmitter Balance. Metabolomic analysis indicated upregulated expression of GABA-related metabolites after CP-9 intervention. This increase in GABA levels likely contributes to enhanced inhibitory neurotransmission, helping to restore excitatory-inhibitory balance in the CNS, which is often disrupted in depression. As the primary inhibitory neurotransmitter, GABA reduces neuronal hyperexcitability and neuroinflammatory responses. CP-9 may enhance GABA synthesis or reduce its metabolic degradation, thereby increasing GABA-mediated inhibitory signaling. This helps restore the excitatory-inhibitory balance in the central nervous system and counteracts neurotransmitter imbalances associated with depression [25,32].

Regulation of Tryptophan Metabolism and the 5-Hydroxytryptamine (5-HT) System. Changes in tryptophan metabolites suggest that CP-9 may promote the conversion of tryptophan to 5-hydroxytryptophan (5-HTP) and 5-HT synthesis while inhibiting the kynurenine pathway shift. As a classical neurotransmitter involved in mood regulation, increased brain 5-HT levels significantly alleviate depressive symptoms. By modulating tryptophan metabolic flux, CP-9 likely contributes to elevated brain 5-HT levels, improving core symptoms such as low mood and anhedonia [33,34].

Restoration of Gut Microbiota Homeostasis and Barrier Function: 16S rRNA sequencing results demonstrated that CP-9 intervention restored gut microecological diversity in depressed mice, increased the abundance of beneficial bacteria such as Lactobacillus and Akkermansia, and reduced levels of potential pathogens (e.g., Escherichia-Shigella). Concurrently, the remodeling of microbial community structure likely promoted thickening of the mucus layer and restoration of intestinal epithelial barrier integrity. This thereby reduced the translocation of gut-derived endotoxins (e.g., lipopolysaccharide, LPS) into the central nervous system via systemic circulation, indirectly mitigating neuroinflammation [35,36].

In summary, *Bifidobacterium animalis* CP-9 exerts significant intervention on depressive-like behaviors through synergistic multi-mechanistic actions:

SCFA-Mediated Anti-inflammatory Effects: CP-9 promotes the synthesis of short-chain fatty acids (SCFAs), such as propionate and butyrate, which not only directly participate in host energy metabolism but also inhibit the release of pro-inflammatory cytokines by activating receptors like GPR41 and GPR43. This suppression of key inflammatory signaling pathways (e.g., NF-κB) alleviates chronic neuroinflammatory responses [37].

GABAergic Regulation and Neurotransmitter Rebalancing: CP-9 modulates γ-aminobutyric acid (GABA) metabolic homeostasis, increasing its central concentration. This enhances inhibitory neuronal signaling, reduces excitotoxicity in the nervous system, restores central neurotransmitter balance, and suppresses the development of anxiety and depressive moods.

Tryptophan Metabolic Reprogramming and 5-HT Pathway Activation: By optimizing tryptophan metabolic flux, CP-9 inhibits inflammation-induced kynurenine pathway activation while enhancing the synthesis and release of 5-hydroxytryptamine (5-HT). This activates cerebral 5-HT pathways, alleviating core depressive symptoms such as anhedonia and low mood at the molecular level [38].

Gut Microecological Reconstruction and Barrier Protection: 16S rRNA sequencing revealed that CP-9 intervention significantly reshapes the gut microbiota ecology, promotes beneficial bacteria (e.g., *Lactobacillus*, *Akkermansia*), reduces pathogen colonization, and enhances intestinal mucosal barrier integrity. This fundamentally attenuates the translocation of gut-derived LPS into the bloodstream, curbing the propagation of peripheral inflammatory signals to the central nervous system [39].

Thus, the antidepressant effects of CP-9 rely not on the regulation of a single pathway but rather on systemic intervention within the “microbiota-metabolism-neuro-immunity” network. This multi-level, network-based mechanism provides a solid theoretical foundation for the application of probiotics in the prevention and treatment of neuropsychiatric disorders. It also offers new research directions and practical pathways for developing functional foods or microecological agents aimed at mood disorder intervention.

## 5. Conclusions

In summary, this study systematically demonstrates that *Bifidobacterium animalis* CP-9 exerts significant antidepressant effects in an LPS-induced murine model of depression. The beneficial outcomes are mediated through multi-faceted actions along the gut-brain axis, as evidenced by the integration of behavioral tests, multi-omics analyses, and pharmacological assessments.

Key experimental findings directly indicate that CP-9 intervention:

(1) significantly modulates the tryptophan-kynurenine metabolic pathway and reduces levels of pro-inflammatory cytokines (e.g., IL-6, TNF-α) in the brain;
(2) restores gut microbiota α-diversity, increases the relative abundance of beneficial short-chain fatty acid-producing bacteria (e.g., *Faecalibacterium*, *Roseburia*), and consequently improves intestinal barrier function;
(3) elicits specific regulatory effects on central GABAergic neurotransmission and mitochondrial energy metabolism, as revealed by non-targeted metabolomics.

These results provide substantive experimental support for the microbiota-gut-brain axis (MGBA) theory and establish a translational medical foundation for developing targeted, strain-specific probiotic interventions against depression. The findings highlight the potential of *Bifidobacterium animalis* CP-9 as a promising psychobiotic candidate for the prevention and adjunct treatment of mood disorders. Future studies are warranted to further validate its precision medicine potential utilizing germ-free animal models and rigorous clinical randomized controlled trials.

## Figures and Tables

**Table 1:** Sucrose preference test results.

Group	Sucrose Preference Index (%)	vs. Model Group (*p*)	Effect Size (ηp^2^)
Blank control	0.78 ± 0.03 **	*p* < 0.001	0.72
Model	0.44 ± 0.05	—	—
Positive control	0.54 ± 0.02 **	*p* = 0.021	0.45
C1 (low dose)	0.53 ± 0.03 *	*p* = 0.038	0.38
C2 (medium dose)	0.60 ± 0.03 **	*p* = 0.007	0.58
C3 (high dose)	0.48 ± 0.03	*p* = 0.312	0.12

Note: * *p* < 0.05 vs. model group; ** *p* < 0.01 vs. model group. Data are mean ± SD. One-way ANOVA with Tukey’s HSD post-hoc test. All groups passed normality and homogeneity tests.

**Table 2:** Forced swim test results.

Group	Immobility Time/Total Time (%)	vs. Model Group (*p*)	Effect Size (ηp^2^)
Blank control	0.28 ± 0.05 **	*p* < 0.001	0.65
Model	0.41 ± 0.04	—	—
Positive control	0.28 ± 0.07 **	*p* < 0.001	0.65
C1 (low dose)	0.37 ± 0.11	*p* = 0.124	0.22
C2 (medium dose)	0.34 ± 0.03 *	*p* = 0.042	0.36
C3 (high dose)	0.27 ± 0.04 **	*p* = 0.003	0.62

Note: * *p* < 0.05 vs. model group; ** *p* < 0.01 vs. model group. Data are mean ± SD. One-way ANOVA with Tukey’s HSD post-hoc test.

**Table 3:** Experimental Results of Inflammatory Factor Detection.

Group	IL-1β (pg/mL)	IL-6 (pg/mL)	TNF-α (pg/mL)
Blank Group	116.56 ± 18.83 **	23.26 ± 2.86 **	163.48 ± 12.14 **
Model Group	376.54 ± 4.33 ##	70.33 ± 1.05 ##	585.36 ± 11.86 ##
Positive Group	168.15 ± 4.7 ##**	24.27 ± 1.27 **	244.36 ± 13.64 ##**
C1 Group	97.12 ± 13.05 **	17.39 ± 1.11 #**	192.46 ± 40.39 **
C2 Group	129.44 ± 15.63 **	23.73 ± 3.04 **	184.22 ± 12.36 **
C3 Group	95 ± 16.32 **	14.89 ± 2.8 ##**	156.73 ± 3.92 **

Note: Compared with the model group, ** indicates a highly significant difference (*p* < 0.01); compared with the blank group, # indicates a significant difference (*p* < 0.05), ## indicates a highly significant difference (*p* < 0.01).

**Table 4:** Statistics of Alpha Diversity Indices.

Sample ID	Feature	ACE	Chao1	Simpson	Shannon	PD_Whole_Tree	Coverage
C1-59	243	244.0805	243.1154	0.8761	4.669	9.9016	0.9999
C1-60	524	528.9675	524.4331	0.9795	6.6461	20.7475	0.9998
C1-62	339	343.4976	340.0784	0.9449	5.2204	22.1052	0.9998
C2-63	376	380.5604	376.8871	0.9566	5.6604	20.771	0.9998
C2-65	392	396.9606	393.5	0.9718	6.1638	15.5563	0.9998
C2-76	288	293.7621	291.25	0.9503	5.1886	24.3267	0.9998
C3-70	418	424.054	420.7273	0.9634	5.824	27.6087	0.9998
C3-72	453	458.0522	453.679	0.964	6.073	26.1185	0.9998
C3-74	431	434.9243	431.8148	0.8964	4.8779	27.1549	0.9998
H1-21	246	251.188	247.5714	0.9354	4.8508	24.937	0.9998

Note: (1) Sample ID: Sample name; (2) Feature: Number of features (OTUs or ASVs); (3) ACE: Abundance-based Coverage Estimator, an index estimating the number of species in a community based on abundance; (4) Chao1: Chao1 index, a metric for measuring species richness; (5) Simpson: Simpson index, an index used to estimate microbial diversity in a sample; (6) Shannon: Shannon-Wiener index, an index used to estimate microbial diversity in a sample; (7) PD_whole_tree: Phylogenetic diversity index calculated based on a phylogenetic tree; higher values indicate greater community diversity; (8) Coverage: Coverage rate of the sample library.

**Table 5:** Differential Metabolite Identification Results.

Name	mz	VIP	log2(FC)	*p* Value	Formula	KEGG
gamma-Aminobutyric acid	104.0707	1.4	−0.5	5.4581E-03	C4H9NO2	C00334
2-Phenylethanol	105.0701	1.3	−0.48	6.1356E-03	C8H10O	C05853
Cytosine	112.0509	1.4	1.87	1.1379E-04	C4H5N3O	C00380
L-Prolinamide	115.0866	1.4	−0.61	1.0088E-03	C5H10N2O	C19781
L-2,4-diaminobutyric acid	118.065	1.4	2.17	4.9064E-03	C4H10N2O2	C03283

Note: Name: Name of the identified substance; mz: Mass-to-charge ratio; VIP: Variable Importance in Projection (VIP) value of the first principal component in OPLS-DA analysis; log2(FC): log2 value of the fold change; *p* value: Statistical *p*-value (a smaller value indicates greater significance of the difference); Formula: Molecular formula of the metabolite; KEGG: KEGG compound identifier.

## Data Availability

The original contributions presented in this study are included in the article. Further inquiries can be directed to the corresponding author.
